# You complete me: tumor cell-myeloid cell nuclear fusion as a facilitator of organ-specific metastasis

**DOI:** 10.3389/fonc.2023.1191332

**Published:** 2023-06-22

**Authors:** Alyssa J. Cozzo, Michael F. Coleman, Stephen D. Hursting

**Affiliations:** ^1^ Duke University School of Medicine, Durham, NC, United States; ^2^ Department of Pathology, Duke University Medical Center, Durham, NC, United States; ^3^ Department of Nutrition, University of North Carolina at Chapel Hill, Chapel Hill, NC, United States; ^4^ Lineberger Comprehensive Cancer Center, University of North Carolina at Chapel Hill, Chapel Hill, NC, United States; ^5^ Nutrition Research Institute, University of North Carolina at Chapel Hill, Kannapolis, NC, United States

**Keywords:** fusion hybrid, cancer metastasis, malignant transformation, immune suppression, organotropism

## Abstract

Every cancer genome is unique, resulting in potentially near infinite cancer cell phenotypes and an inability to predict clinical outcomes in most cases. Despite this profound genomic heterogeneity, many cancer types and subtypes display a non-random distribution of metastasis to distant organs, a phenomenon known as organotropism. Proposed factors in metastatic organotropism include hematogenous versus lymphatic dissemination, the circulation pattern of the tissue of origin, tumor-intrinsic factors, compatibility with established organ-specific niches, long-range induction of premetastatic niche formation, and so-called “prometastatic niches” that facilitate successful colonization of the secondary site following extravasation. To successfully complete the steps required for distant metastasis, cancer cells must evade immunosurveillance and survive in multiple new and hostile environments. Despite substantial advances in our understanding of the biology underlying malignancy, many of the mechanisms used by cancer cells to survive the metastatic journey remain a mystery. This review synthesizes the rapidly growing body of literature demonstrating the relevance of an unusual cell type known as “fusion hybrid” cells to many of the hallmarks of cancer, including tumor heterogeneity, metastatic conversion, survival in circulation, and metastatic organotropism. Whereas the concept of fusion between tumor cells and blood cells was initially proposed over a century ago, only recently have technological advancements allowed for detection of cells containing components of both immune and neoplastic cells within primary and metastatic lesions as well as among circulating malignant cells. Specifically, heterotypic fusion of cancer cells with monocytes and macrophages results in a highly heterogeneous population of hybrid daughter cells with enhanced malignant potential. Proposed mechanisms behind these findings include rapid, massive genome rearrangement during nuclear fusion and/or acquisition of monocyte/macrophage features such as migratory and invasive capability, immune privilege, immune cell trafficking and homing, and others. Rapid acquisition of these cellular traits may increase the likelihood of both escape from the primary tumor site and extravasation of hybrid cells at a secondary location that is amenable to colonization by that particular hybrid phenotype, providing a partial explanation for the patterns observed in some cancers with regard to sites of distant metastases.

## 1 Introduction

### 1.1 Every cancer is biologically unique

More than 200 types of cancer have been identified and subcategorized based on factors such as tissue of origin, cell of origin, presence of specific driver mutations, histopathologic morphologies, immunophenotype, response to existing therapies, and many more. Despite our best efforts as clinicians and cancer researchers, this biological complexity has impeded the identification of new cancer therapies that consistently retain efficacy across multiple cancer types and subtypes. Intratumoral heterogeneity, accumulation of new molecular aberrations, clonal evolution with development of therapeutic resistance, and interaction with each individual’s unique immunophenotype further obfuscate the predictive and/or prognostic potential of currently available biomarkers, resulting in the potential for very different treatment outcomes even in two patients with the same subtype of cancer.

Transformative work by Hannahan, Weinberg, and others has identified specific “hallmarks” that together define cancer as a disease entity (1–3). Of these, the hallmark of cancer metastasis to distant organs is the primary cause of cancer-associated morbidity and mortality (see **Table 1**). Remaining gaps in our mechanistic understanding of which cancer cells will ultimately metastasize present an obstacle to improving patient outcomes. In particular, the processes allowing metastasizing cancer cells to evade immune surveillance while surviving the hydrodynamic and metabolic stresses of the circulatory system remain both a compelling mystery and a potentially rich source of therapeutic targets.

**Table 1:** Approaches to identification of tumor cell-macrophage hybrid cells in human cancers.

Study	Cancer type	Sample type	Cell Population(s) and Study Definition(s)
Shabo et al. *Int J Cancer.* (4) (1); Shabo et al. *Int J Cancer.* 2009 (2)	Breast cancer, rectal cancer	Primary tumor	Fusion hybrids: CD163, MAC387, or CD68 expression in cells with malignant morphology
Ramakrishnan et al. *Cancer Res* (5) (3).	Serous epithelial ovarian cancer	Ascitic fluid of cytopathologically confirmed cases	Fusion hybrids: EpCAM+CD45+; CA125+CD45+
LaBerge et al. *PLoS One* (6) (4).	Melanoma	Primary tumor and nodal metastasis	Fusion hybrids: Donor-patient hybrid genome following BMT, assessed via short tandem repeat (STR) length-polymorphisms
Yilmaz et al. *Bone Marrow Transplant* (7) (5).	Renal cell carcinoma	Primary tumor	Fusion hybrids: Combined H&E staining and dual-label FISH used to detect carcinoma cells containing both a Y chromosome (donor marrow) and 3+ copies of chromosome 17 (recipient RCC)
Chakraborty et al. *Bone Marrow Transplant* (8) (6).	Renal cell carcinoma	Metastatic lesion	Fusion hybrids: presence of donor allele *A* in tumor cells microdissected from renal cell carcinoma metastasis in a genotype *OO* bone marrow transplant recipient
Gast, et al. *Sci Adv* (9) (7).	Pancreatic adenocarcinoma	Peripheral blood, flow/FACS	CHCs: cytokeratin (CK)+CD45+; EPCAM+CD45+ These cells also expressed: CD163, CD68, CSFR1, CD66b, CD14, CD16, CD11c, Muc4 CTCs: CK+CD45−
Aguirre, et al. *Oncoimmunology* (10) (8).	Lung adenocarcinoma	Peripheral blood, primary and metastatic tumor	Fusion hybrids: EPCAM+CD45+ CD36+CD14+PANK+ CHCs: EPCAM+CD45+ CD36+CD14+PANK+ CTCs: EPCAM+CD45-
Clawson et al. *PLoS One* (11) (9).	Pancreatic adenocarcinoma	Cultured cells isolated from peripheral blood	CHCs: Co-expression of MIF + ZG16B, CD163 + Pan-CK CD206 + ZG16B CD204 + ZG16B CD204 + S100BPB
Clawson et al. *PLoS One* (12 *).*	Melanoma	Peripheral blood	CHCs: Dual immunofluorescent staining for melanocytic and macrophage markers, respectively ALCAM + CD204 pan-Cytokeratin + CD204 EpCAM + CD206 MLANA + CD204 MLANA + CD206
Dietz et al. *Sci Rep* (13) (10).	Carcinoma, uveal melanoma, glioma, and pancreatic neuroendocrine tumors	Peripheral blood	CTCs: identified by protein expression of canonical tumor markers (CK+; NKI/beteb+; GFAP+; or chromogranin A + synaptophysin, respectively) CHCs: identified as cells co-positive for a tumor protein and CD45

Definitions used to identify circulating hybrid cells (CHCs), circulating tumor cells (CTCs), and fusion hybrids in peripheral blood and tumor tissue of patients with cancer include cells either co-expressing epithelial and leukocyte antigens or tumor cells bearing DNA from both donor and recipient following allogeneic BMT transplant. In this table, the term “fusion hybrids” refers to cells found within a primary or metastatic tumor, as opposed to CHCs and CTCs cells collected from peripheral blood.

### 1.2 Organotropism in cancer metastasis

Although propensity for metastasis and primary sites of metastatic lesion development vary across different cancer types, cancers arising from the same tissue-of-origin may exhibit a non-random distribution of spread, a phenomenon known as ‘‘organotropism’’ or organ-specific metastasis. For example, breast cancers tend to metastasize to the liver, bones, lungs/pleura, and brain, while prostate cancers predominantly show metastatic spread to bone, with a considerably lower frequency of metastasis to other anatomical sites (14) (**Table 1**). The process of distant metastasis requires successful completion of several steps, including tissue invasion at the primary tumor site, intravasation, survival in circulatory transit, extravasation, and colonization of the secondary site. During this process, cancer cells must survive obstacles such as anoikis, immune attack, shear stress, and metabolic stress. Moreover, there is typically a lack of pro-survival and growth signals in both the hematogenous/lymphatic circulation and the organ site at which they extravasate. Considering the stochastic nature of biology and the staggering number of variables encountered along the metastatic cascade, how is it that we see these patterns in cancer spread?

Proposed explanations for metastatic organotropism include hematogenous versus lymphatic dissemination; the circulation pattern of the tissue of origin; tumor-intrinsic factors such as particular driver mutations; pre-existing organ-specific microenvironments; long-range induction of premetastatic niche formation (e.g., via tumor- and immune cell-derived exosomes); and interactions between tumor cells and the host secondary tumor microenvironment that promote colonization after extravasation (so-called “prometastatic niches”) (15, 16). An example in which differential circulation pattern seems to be the primary determinant of metastasis pattern would be cancers of the cancers of the distal colon and proximal rectum, which most commonly metastasize to the liver, versus cancers of the distal rectum and anus, which show a higher incidence of metastasis to lung (17). Despite their anatomical proximity, the route of venous blood from these two sites to the lungs is very different: the colon and proximal rectum must first pass through the hepatic portal system before reaching the pulmonary circulation, while blood drained from the distal rectum bypasses the portal system via the internal carotid veins and instead travels directly to the lungs (17).

While circulatory pattern may largely explain the difference in major site of metastatic lesion development for proximal versus distal rectum, in other cases the reasons for metastasis site pattern are less straightforward. Importantly, the point in tumor progression and evolution at which tropism of a given cancer cell is determined remains unknown. It is possible that a subset of primary tumor cells acquires specific properties at their initial site that allow them to reach specific secondary sites that are hospitable only to cancer cells with those specific properties. Alternatively, tumor cells may simply shed from the primary site indiscriminately, leaving the circulation and secondary tissue microenvironment as the major determinants of site of eventual lesion development. It is likely that all of the factors listed above in some way influence the pattern of metastatic distribution for a given cancer type.

## 2 Fusion hybrids: nuclear fusion and malignant transformation

Increasing evidence supports homotypic or heterotypic cellular fusion as an important process in cancer biology. Indeed, the century-old cancer cell-leukocyte fusion theory of metastasis views the acquisition of a metastatic phenotype as a secondary feature imposed on a primary tumor cell via fusion with a healthy migratory leukocyte such as a macrophage (18). There is also strong evidence for cell-cell fusion as a contributor to intratumoral heterogeneity (19–23). Heterotypic fusion of tumor cells with infiltrating immune cells may allow for rapid gain of discrete cellular behaviors associated with malignancy, including acquisition of migratory and invasive phenotypes, the ability to travel “incognito” in circulation, and an increased ability to extravasate at sites of inflammation via expression of leukocyte-restricted proteins involved in immune cell trafficking. This section will discuss the empirical evidence for *in vivo* epithelial-immune cell fusion as a driver of tumorigenesis and tumor heterogeneity. Subsequent sections will address tumor cell-immune cell fusion in acquisition of malignant potential and metastatic organotropism.

Cell-cell fusion is a well-established physiological process involved in mesenchymal cell differentiation as well as fertilization, placentation, myogenesis, osteogenesis, wound healing and tissue regeneration (20, 23, 24). Additionally, fusion between hematopoietic and non-hematopoietic cells has been detected in response to inflammation and injury in Purkinje neurons, hepatocytes, cardiomyocytes, skeletal muscle cells, and intestinal stem or progenitor cells (25). For example, while fusion of intestinal epithelium and circulating bone marrow-derived cells does occur outside of the context of injury, basal rates of intestinal epithelium and circulating bone marrow-derived cells are increased 2 fold by inflammation and epithelial proliferation in response to injury, highlighting that the regulation of fusion events is dynamic and inducible (26).

Increased fusion in response to inflammation is noteworthy considering that an inflammatory and hyperproliferative microenvironment is a risk factor for, and characteristic of, development of solid tumors. Given the importance of cell-cell fusion in wound healing, it is therefore unsurprising that reports of cell-cell fusion in the context of cancer are increasing (9, 10, 23, 24, 27–32). Indeed, cells co-expressing immune and neoplastic/epithelial markers (referred to hereafter as *fusion hybrid cells*) have been detected in breast (4, 33), colorectal (34), pancreatic (9, 11), ovarian (5), and renal cell carcinomas (7, 8) as well as melanoma (5, 6, 35). At this time, it is unclear what triggers heterotypic cell fusion in tumors, nor is it well understood how a fusion partner is selected. However, Pawelek and Chakraborty have published several studies demonstrating tumor cell-leukocyte fusion as an important source of myeloid traits in cancer (18, 31).

### 2.1 Heterotypic nuclear fusion results in massive genome rearrangement, chromosomal instability, aneuploidy, and profound tumor heterogeneity

**Figure 1:**
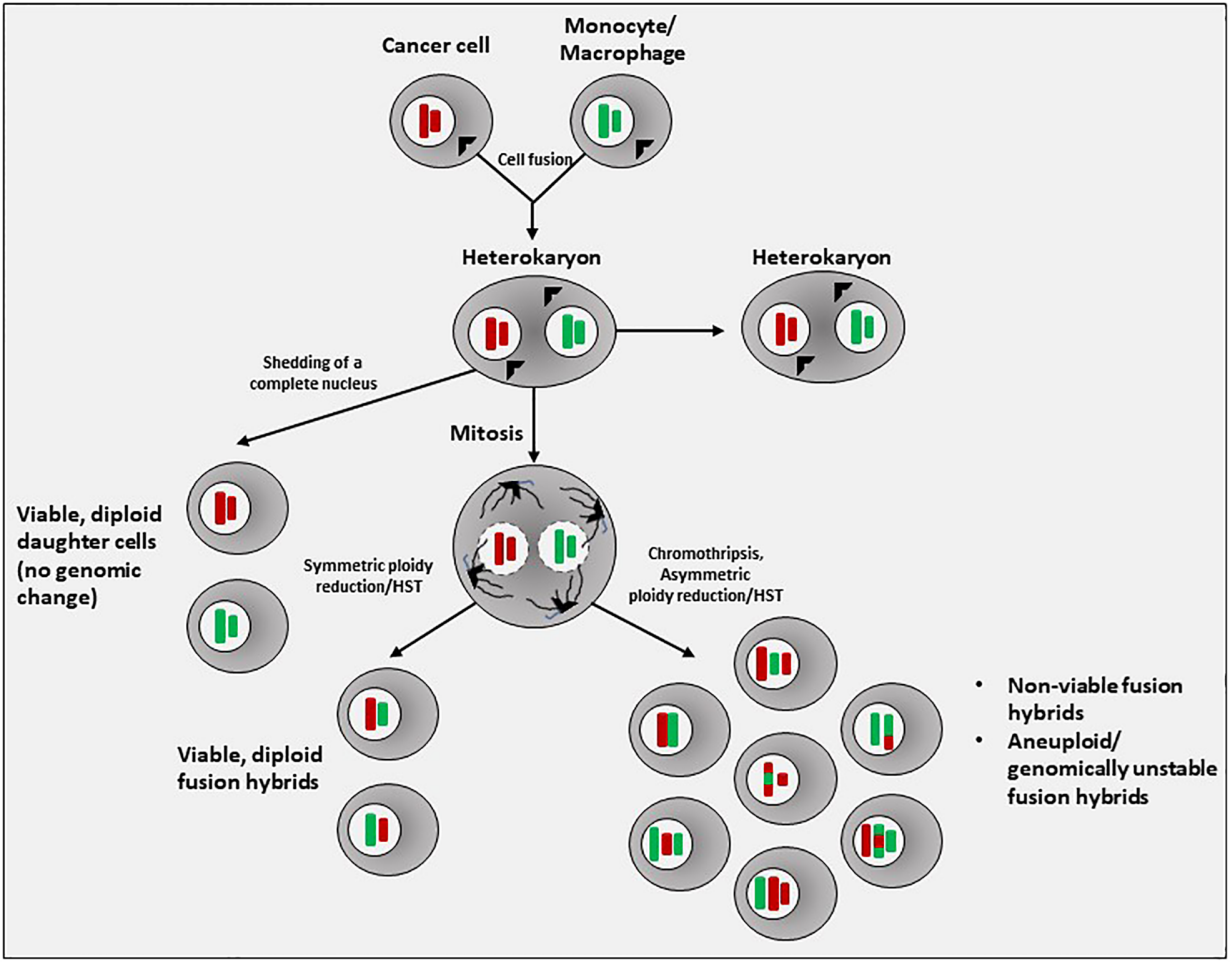
Generation of fusion hybrids. In the most often observed process of tumor cell-immune cell heterotypic cell fusion, a cancer cell and a monocyte or macrophage first fuse to form a cell with multiple (>2) nuclei of different lineage, known as a heterokaryon. If nuclear fusion does not occur, a heterokaryon can simply shed an entire nucleus. If nuclear fusion does occur, a synkaryon is formed. The fused nucleus will initially contain the complete chromosomal content of both fusion partners (4N). In the subsequent process of mitotic division and ploidy reduction, chromosomes may be lost, damaged, undergo translocations, undergo chromothripsis and reassembly, or simply be re-sorted, resulting in a random segregation of parental alleles. These processes introduce aneuploidy and genomic instability, resulting in nonviability, senescence, or possibly malignancy. Thus, the stochastic nature of ploidy reduction in fusion hybrids results in a highly heterogeneous population of daughter hybrid cells.

Cells formed by the fusion of disparate cell types and containing multiple discrete nuclei of heterologous origin are called *heterokaryons*, while the term *synkaryons* refers to a cell with a single nucleus that was formed by two pre-existing nuclei following cell-cell fusion (20). Cell-cell fusion first results in the production of bi- or multinucleated hybrid cells (heterokaryons) (**Figure 1**). The presence of only one nucleus following cellular fusion requires either the shedding of an intact nucleus or fusion of nuclei within a heterokaryon, which can either remain as a heterokaryon or undergo heterokaryon-to-synkaryon transition (HST) and ploidy reduction, giving rise to mononucleated daughter cells containing chromosomal DNA from both fusion partners (36) (**Figure 1**).

Possible outcomes of cell-cell fusion followed by sorting and the selective loss of chromosomes to allow for continued cell viability (i.e., HST) include either daughter cells with a euploid/diploid karyotype or aneuploid daughter cells that are genomically unstable, the latter of which may resemble the instability seen in transformed, malignant cells. Indeed, cell fusion generates aneuploidy, chromosomal instability, and DNA damage that result in phenotypic heterogeneity, transformation, and the capacity to form tumors (19–23).

The mechanisms of random merging of heterotypic parental chromosomes are complex and still not well understood. However, a first mitotic division is necessary for nuclear fusion to occur, yielding a single nucleus containing DNA from both parental cells (9). During the merging of genomes, the phenomenon known as *chromothripsis* may occur (chromo – referring to chromosomes; -thripsis meaning “to shatter into pieces”) (37). Chromothripsis is a large-scale mutational process that occurs through massive genomic rearrangement during a single catastrophic event within a cell. In the process of chromothripsis, chromosomes inside of micronuclei are scattered into tens to thousands of DNA fragments, then reassembled in random order, giving rise to derivative chromosomes with extensive rearrangement. Evidence for the possibility of chromothripsis during nuclear fusion is provided by Delespaul et al., who reported heterogeneous genomes among clones generated via fusion of two non-transformed but immortalized fibroblast cell lines, resulting in growth of tumors with massive genomic alterations similar to those of human pleomorphic mesenchymal tumors (23). Similar findings of a highly rearranged genome after fusion of fibroblast cell lines were reported by Briti et al., who also noted metabolic reprogramming in fusion hybrids, including much greater overall metabolic activity and enhanced glycolysis resembling Warburg metabolism (27).

Given the staggering number of possible genome combinations generated via HST/ploidy reduction, it is likely that many of the resulting fusion hybrids do not survive. A post-hybrid selection process (PHSP) after cell-cell fusion has been proposed, wherein selective pressures within the microenvironment ensure survival of only those hybrid daughter cells that are able to overcome the resulting genomic instability and re-establish cellular and metabolic functionality (38, 39). Importantly, HST/ploidy reduction and the PHSP occur in a unique manner in each hybrid cell (40), creating hybrid clones with highly variable phenotypes (22) (**Figure 1**). Zhou et al. reported that homotypic cell fusion between non-malignant intestinal epithelial cells not only initiated malignancy, but the heterogeneity among the resultant hybrids also directed tumor evolution (22). Passaging of individual hybrid clones resulted in a subset of clones in which contact inhibition was lost and cells gained the ability to undergo anchorage-independent growth, defining features of cellular transformation.

Despite the potential for ongoing chromosomal instability in the process of tumor formation, karyotypic analysis showed retention of the original hybrid clonal karyotypes in tumors grown within immunodeficient hosts (22). Interestingly, while growth rates and histologic phenotype were similar across tumors generated from the same original hybrid clone, tumors from different clones exhibited distinct rates of growth as well as different degrees of invasiveness and glandular differentiation. The authors concluded that the retention of distinct and stable properties in tumors generated by different fusion hybrid clones indicated that heterogeneity across clones was established during the fusion event or in early passages, rather than by ongoing genomic evolution during tumor development.

### 2.2 Among immune cells, monocytes/macrophages are the predominant tumor cell fusion partner

While homotypic cell fusion does appear to result in acquisition of new cellular phenotypes, aneuploid cells generated by homotypic fusion cannot be distinguished from cells in which aneuploidy has arisen via other processes (24). Additionally, given identical genomes and similar exomes in homotypic fusion partners, HST/ploidy reduction to a diploid cell complicates detection of the initial fusion product or the clonal progeny of this fusion in human subjects. Recognizing this possibility, Weiler and Dittmar have proposed the “dark matter hypothesis of cell fusion”, wherein malignant cells produced via homotypic cell fusion and subsequent ploidy reduction are termed “dark matter hybrids”, and their prevalence and relevance in tumor biology is believed to be grossly underestimated (24).

Hybrids generated by heterotypic cell fusion, on the other hand, are more easily detectable. Approaches to identifying fusion hybrid cells *in vitro* and *in vivo* have varied across a number of elegant studies, and include size and flow cytometric scatter properties, co-expression of nuclear and/or cytoplasmic fluorescent proteins, and karyotype analyses following fusion of cells bearing different sex chromosomes (e.g., “male” immune cells [XY] fused to neoplastic cells with [XO]) (9, 22). Heterotypic fusion with immune cells *in vivo* is detectable through co-expression of lineage markers of different cell lineages. Using these approaches, several studies have reported that fusion between epithelial cells and monocytes or macrophages happens readily; however, fusion with cells of lymphocytic lineage is rare (9, 10, 12, 28, 29).

#### 2.2.1 *In vitro* generation of macrophage-tumor cell fusion hybrids

Aguirre et al. conducted fusion assays using human lung cancer cell lines and different human leukocyte lineages isolated from buffy coats of peripheral blood samples (10). Following culture under cancer stem cell (CSC) conditions, lung cancer cells were co-cultured with human monocytes, macrophages, neutrophils, or lymphocytes. While monocytes and macrophages were equivalent in fusion efficiency, lymphocyte and neutrophil co-cultures did not result in hybrids. Additional studies implicated the scavenger receptor CD36 as a possible mediator of increased fusion between tumor cells and monocytes/macrophages, as upregulation of CD36 in both CSCs and monocytes, induced by exposure to 4-hydroxynonenal, significantly increased hybrid formation. These findings were further validated using overexpression, knockout, and rescue experiments with CD36, which showed a strong correlation between CD36 and fusion events (10). Activation of macrophages to an M2-like phenotype via IL-4, metabolic manipulation, or oxidized LDL exposure also resulted in increased fusion rates, whereas monocytes activated using IFN-gamma showed fusion rates that closely matched that of controls. RNA-seq analyses of fusion hybrids – defined in this study as CD14+ cells also expressing CD36 and positive for pan-cytokeratins (PANK) – revealed expression of transcripts associated with cancer and expressed primarily in parent lung cancer cells, as well as transcripts involved in immune-associated pathways and expressed primarily in parent monocytes/macrophages.

Fusion hybrids generated via co-culture of MC-38^H2BmRFP^ colon cancer cells and GFP-expressing macrophages were also able to functionally divide into viable daughter cells expressing both reporter proteins and bearing a transcriptional profile characteristic of both parental predecessors yet also exhibiting new, unique characteristics (9, 28). Independent analysis comparing gene expression across five hybrid clones demonstrated a high degree of heterogeneity in macrophage gene expression, consistent with previous reports of heterogeneity among hybrids (9). Subsequent subcutaneous flank injection of colon cancer-macrophage fusion hybrids into syngeneic immunocompetent mice revealed shorter doubling times *in vivo* than those seen in tumors from the unfused parent MC38 cell line (9).

#### 2.2.2 Evidence for macrophage-tumor cell fusion *In vivo*

A variety of approaches have also confirmed *in vivo* generation of fusion hybrid cells, both in animal models (i.e., mice) and in human subjects with known cancers. Gast et al. injected RFP-labeled B16F10 melanoma cell lines into actin-GFP and R26R-YFP Cre reporter mice to determine whether dual-positive fusion hybrids could be formed *in vivo* (9). Indeed, rare hybrid cells were detected within primary tumors of both models (RFP+/GFP+ fusion hybrids in actin-GFP recipient mice and RFP+/YFP+ fusion hybrids in R26R-YFP Cre reporter mice). RFP+/YFP+ hybrids comprised an average of 0.48% of RFP+ cells, with a range of 0.03-0.69%. Double-positive hybrid cells were subsequently isolated using FACS and injected intradermally into secondary recipient mice, again resulting in generation of tumors. Interestingly, in additional experiments using hybrid clones generated using the MC38 colon cancer cell line, fusion hybrids showed a greater faster average rate of *in vivo* tumor growth, but also greater variability in rates of tumor growth, than was seen in tumors generated from the unfused MC38 parental cancer cell line (9).

To confirm whether monocytes/macrophages were also the primary fusion partner *in vivo*, as seen *in vitro*, B16F10 melanoma cells expressing an fl-dsRed-fl-eGFP allele were orthotopically injected into LysM-Cre transgenic mice (9). Both primary tumors and lung metastases harbored hybrid cells, defined in these experiments as cells expressing both the melanocytic lineage-specific transcription factor MITF and Cre-mediated GFP.

Identification and isolation of *in vivo*-derived macrophage-epithelial fusion hybrids have also been performed using parabiosis models (28). Powell et al. reported *in situ* hybridization of macrophages and intestinal cells, with fusion hybrids continuing to reside within the intestinal epithelial compartment and retaining an epithelial phenotype, yet also expressing the macrophage gene F4/80 shortly after fusion occurred. Interestingly, although F4/80 in macrophages is a cell surface protein, in the intestinal epithelium-macrophage fusion cells it appeared to be localized to the cytoplasm for a period of around 4 weeks, after which expression of the F4/80 protein was lost, but mRNA transcripts were still detectable. Transcriptome analyses of FACS-isolated unfused intestinal epithelial cells, unfused macrophages, and intestinal cell-macrophage fusion hybrids showed that a subset of the transcripts differentially regulated across the three groups were uniquely expressed in fusion hybrids (28). Karyotype analyses of single hybrid cells in each of these two studies again revealed variable chromosome numbers, further indicating that heterotypic cell fusion may contribute to tumor heterogeneity (9, 28).

As mentioned previously, identification of fusion hybrids in human subjects poses a challenge. Several groups have used co-expression of epithelial and hematopoietic markers (5, 12, 34). While this strategy for identifying putative macrophage-epithelial fusion hybrids in human cancer biopsies is a relatively simple approach, it cannot be ruled out that acquired expression of macrophage-like antigens may simply be due to the genomic instability intrinsic to cancer cells (though the probability of random activation of multiple myeloid-specific genes in multiple cancer cells in a primary tumor is low). An alternative option is analysis of tumor biopsies from female cancer patients who have previously received a sex-mismatched (XY) bone marrow transplant and subsequently developed a secondary solid tumor. Studies using this approach have reported the presence of neoplastic cell nuclei containing a Y chromosome in pancreatic ductal adenocarcinoma, renal cell carcinoma, head and neck squamous cell carcinoma, and lung adenocarcinoma (7–9).

Collectively, these data strongly support that multiple cancer types are capable of spontaneously forming fusion hybrids *in vivo* and confirm that macrophage-cancer cell fusion specifically does occur *in vivo*. Additionally, *in vivo* HST/ploidy reduction also appears to generate a heterogeneous hybrid population, at least a fraction of which retain tumorigenicity and exhibit accelerated tumor progression relative to unfused parent cancer cell lines. Thus, fusion hybrids produced from heterotypic cell fusion between macrophages and carcinoma cells may contribute to both tumorigenesis and intratumoral heterogeneity. The predominance of macrophages as a fusion partner is unsurprising, given both their fusogenic nature and their prevalence in solid tumors (41–43). These findings also add yet another role to a large body of literature demonstrating tumor-educated macrophages to be critical players at every stage of metastatic progression (41–43). However, the mere presence of fusion hybrids within primary tumors and metastatic lesions does not *per se* implicate fusion hybrids as participants in the actual metastatic process.

## 3 Macrophage-tumor cell fusion in acquisition of metastatic potential

Among the events of the metastatic cascade, the mechanisms whereby a subset of tumor cells acquire the ability to escape into circulation and evade immune destruction in transit may be the least understood. This is in part due to the lack of a validated genetic signature identifying or defining the population capable of metastasis formation (44). Unfortunately, this knowledge gap also precludes precise therapeutic targeting of this subset of tumor cells within the primary tumor. The most widely accepted hypothesis currently for inception of metastatic capacity is the induction of an epithelial-to-mesenchymal transition (EMT) in a subset of cancer cells within the primary tumor, which confers increased motility and invasiveness that facilitate their escape into the bloodstream (45). Induction of EMT has been attributed to signaling between cancer cells and neighboring stromal cells, including fibroblasts and myeloid cells (46). For this to occur, two features of the carcinoma cells are implied: 1) that they are intrinsically responsive to EMT-inducing signals, and 2) that they are primed in such a way as to allow for activation of latent EMT programs (45).

Notably, similar changes in cell behavior are observed following cancer cell-macrophage fusion hybridization; indeed, some have even suggested that EMT might better be described as an “epithelial-myeloid transition” (29). Colon cancer-macrophage fusion hybrids displayed enhanced motility and migration relative to the unfused parent colon cancer cell line, along with increased expression of factors associated with stemness (9). Similar findings were found using human lung cancer-macrophage fusion hybrids, with hybrid cells again showing increased proliferation as well as enhanced migration and invasiveness compared to controls (10).

In experimental metastasis assays, MC38-derived fusion hybrids injected into spleens resulted in increased metastatic foci in liver per area compared to unfused parental cancer cells (9). Likewise, B16F10-derived fusion hybrids injected intravenously formed metastatic lung lesions of a greater tumor area than those formed following injection of unfused B16F10 cells. Consistent with these findings, gene expression analyses showed upregulation of pathways contributing to tumor invasion—e.g., attachment, matrix dissolution, and migration— in hybrids relative to unfused tumor cells.

Altogether, fusion hybrids in these studies displayed increased features consistent with metastatic capability, including motility, invasiveness, and chemotaxis, compared to unfused cancer cells. Experimental metastasis assays bypass the initial steps of metastasis, and therefore only assess the ability of tumor cells to survive in circulation, arrest, extravasate, and grow in a particular organ following intravenous injection. With that said, observation of more extensive metastatic lesions in mice injected with fusion hybrids (9) does suggest an enhanced ability to complete these later steps of the metastatic cascade.

### 3.1 Fusion hybrids among circulating tumor cells

A requirement for metastasis initiation is successful dissemination from the primary tumor to the peripheral blood and eventually a distant organ site. Two cell populations that are strongly implicated as having metastasis-initiating potential include circulating tumor cells (CTCs) and disseminated tumor cells (DTCs), often identified as cells expressing epithelial markers yet found in the blood or bone marrow, respectively, of patients with carcinoma (47). CTCs are strongly implicated in initiation of subsequent metastatic lesions (48), as their levels have been shown to be an accurate and independent predictor of progression-free and overall survival in multiple cancer types (49–51). However, it appears that only a rare subset of conventionally-defined CTCs possesses malignant traits indicative of metastatic potential (52).

Additionally, the CTC population may be more biologically heterogenous than is currently recognized in the majority of studies in this area. The classical definition of CTCs in human cancer is a circulating cell expressing a tumor antigen (usually a cytokeratin) *and not expressing the pan-leukocyte antigen CD45* (48–52). This definition is problematic for at least two reasons: 1) the process of EMT involves at least partial loss of epithelial traits, and 2) it guarantees exclusion of additional circulating populations that are positive for both tumor markers and markers of other cell lineages from routine analyses (e.g., a CD45+ circulating hybrid cell (CHC) population). Aguirre et al. used a CD14+CD36+PANK+ macrophage-lung cancer fusion hybrid signature, developed through a combination of FACS isolation and RNA-seq, to assess whether macrophage-cancer cell fusion hybrids could be found in peripheral blood of patients with lung cancer (10). Though triple-positive cells were not detected in control subjects, their presence in the blood of patients with lung cancer correlated to primary tumor size, spread to lymph nodes, and clinical stage. A significant correlation was similarly observed between CD14/CD36/PANK co-localization in lung samples and metastasis occurrence. Interestingly, triple-positive cells were found only in the lungs of those patients who later developed metastases (present in 60% of the primary tumors of patients who subsequently developed metastasis, and in 80% of metastatic lesions). In fact, among those patients whose primary tumor was positive for cells bearing the CD14/CD36/PANK signature, the frequency of patients who did not later develop metastasis was zero.

Despite their low abundance within primary tumors (9), CHCs appear to dramatically outnumber CTCs in blood samples across a wide spectrum of malignancies, including both epithelial and non-epithelial cancers (9, 13). These findings support the ability of fusion hybrids not only to complete the first few steps of the metastatic cascade, but to do so in a more efficient manner than traditional, unfused CTCs. Inclusion of cells expressing both epithelial and leukocyte- or myeloid-specific markers in studies investigating the importance of circulating cancer cells may demonstrate CHCs to be additional important effectors of metastatic spread (12)

### 3.2 Fusion hybrids potently suppress immune response to evade immunosurveillance

The consistent finding that circulating fusion hybrids outnumber conventional CTCs also begs the question of whether fusion hybrids interact differently with the immune system than unfused cancer cells. In cytokine exposure assays, transforming growth factor (TGF-beta 1-3) induced dose-dependent suppression of MC38 proliferation, but showed no effect on hybrids (9). More strikingly, hybrids were resistant to tumor necrosis factor–alpha (TNF-alpha), which profoundly inhibited proliferation of MC38 cells. Lung cancer fusion hybrids also show some ability to both modulate and overcome immune surveillance (10). Exposure of CD4+ and CD8+ T-cells to fusion hybrids markedly reduced mitogen-induced proliferation of both T cell populations. However, hybrids did induce upregulation of FoxP3, PD-1, and CTLA4 expression by T cells, which was not observed in co-culture with lung cancer stem cells. Additionally, NK cells co-cultured with fusion hybrids showed reduced perforin production and cytotoxicity compared to NK cells co-cultured with unfused lung cancer stem cells (10). The latter finding may have been mediated by higher expression of PD-1, CD39, CD73 and SIGLEC5 by fusion hybrids, as this effect was reverted in the presence of inhibitory antibodies to these proteins (10). Similarly, co-culture with fusion hybrids led to a smaller up-regulation of IFNγ, TNFα and IL-6 production in peripheral blood mononuclear cells (PMBCs). While these data are limited, they suggest that fusion hybrids may be more resistant to innate mechanisms of tumor suppression and elicit a lesser anti-tumor response in leukocytes of multiple lineages.

## 4 Organotropism in fusion hybrids: enhanced extravasation at metastatic niches

The term “premetastatic niche” was coined to describe a microenvironment in a secondary organ site that has been rendered permissive to metastatic outgrowth in advance of cancer cell entry through the activity of circulating factors released in a variety of forms by the primary tumor (53). These tumor-derived factors have been shown to induce myeloid cell recruitment to sites such as lung and liver, which subsequently prepare a hospitable environment for later colonization via release of various chemokines, inflammatory mediators, growth factors, and angiogenic factors (54). In particular, the establishment of an inflammatory milieu at a secondary site, either prior to or coincident with the arrival of circulating cancer cells, facilitates seeding, survival, and proliferation of tumor cells. Additional resident cell types within the pre-metastatic niche, such as fibroblasts (or stellate cells in the liver) and endothelial cells, also release inflammatory cytokines, recruiting additional waves of myeloid cells (54). For example, in lung premetastatic niches, secretion of the pro-inflammatory mediators S100A8/A9 induces the expression of serum amyloid A (SAA), which recruits myeloid cells to these sites and activates them to an inflammatory state via TLR4 (55). These myeloid cells subsequently promote the migration of primary tumor cells to the secondary lung sites by enhancing pre-metastatic niche formation through expression of proinflammatory cytokines and extracellular matrix remodeling (55). In particular, macrophages are major players in establishment of the pre-metastatic niche as well as tumor cell arrest at the secondary site, tumor cell extravasation, and successful outgrowth at metastatic sites (43, 54).

### 4.1 Fusion of cancer cells with monocytes/macrophages may expand the options for extravasation

Extravasation and adaptation of newly arrived cancer cells to the tissue microenvironment of distal organs are stringent rate-limiting steps in metastasis. Although we are gaining a better understanding of how premetastatic niches are prepared, it is still unclear how or why they are the sites selected. This section will discuss the available evidence for acquisition of specific leukocyte chemokine receptor and/or cellular adhesion molecule expression by tumor cells as a possible contributor to metastatic organotropism.

Leukocyte homing and trafficking are essential functions of both innate and adaptive immune responses. The basic molecular processes involved in trafficking are chemotaxis and cellular adhesion. While chemotaxis is mediated mainly by chemokines, cellular adhesion is mediated by selectins and integrins and their interactions with their respective receptors. Chemokine signaling through chemokine receptors is also a powerful physiologic activator of integrins via “inside-out” signaling, which regulates the ligand-binding affinity of the cell surface receptors in response to changes in the environment (56). Expression of select chemokines and integrin ligands by the local endothelium varies based on tissue type and current conditions (e.g., inflammation). This dynamic pattern of receptor expression creates localization signals, ensuring that leukocyte populations are able to traffic to and extravasate at the sites and times that they are needed. For example, local inflammation generates potent signals for recruitment of myeloid cells, including expression of specific chemokines and upregulation of cell adhesion molecules by the associated endothelium. It has been suggested that tumor cells may use similar localization signals for extravasation and colonization at metastatic sites.

In chemotaxis assays, fusion hybrids migrated toward colony-stimulating factor 1 (CSF1) or stromal cell-derived factor-1 (SDF1) at multiple concentrations, whereas unfused MC38 cancer cells showed no response to either chemoattractant (9). Increased migration towards SDF1 in particular has implications for organotropism, as the CXCL12 (SDF-1)/CXCR4 axis plays a pivotal role in bone metastasis (57). However, it should be acknowledged that this particular finding was investigated in in a colorectal cancer cell line (9), which very rarely metastasizes to bone (58).

Interestingly, experimental metastasis studies in mice inoculated with non-small cell lung cancer stem cells versus monocyte-lung cancer cell hybrids found hybrid cells, but not unfused parental lung cancer stem cells, in lymph nodes and spleens up to 28 weeks after tail vein injection (10). Similarly, when CD36+CD14+PANK+ subpopulations isolated from lung-cancer patients were injected into mice, these cells were also found in spleens after 28 weeks, along with unstructured morphology and high levels of TTF-1. While circulatory pattern may largely explain organotropism in some cancer types (e.g., colorectal cancer metastasizing to liver), metastasis to the spleen by primary lung cancer is not consistent with the circulatory pattern hypothesis of organotropism (13).

### 4.2 Leukocyte-restricted cell adhesion molecule expression in fusion hybrids

As extravasation at two of the major sites for metastases – liver and lung – appears to be primarily selectin-independent (56), this final section will focus on expression of leukocyte-restricted integrins by tumor cells. Integrins are cell adhesion molecules consisting of two non-covalently associated α and β chains and a single transmembrane domain. The most important groups within the integrin receptor family for leukocyte arrest are the β1 and β2 integrins; β2 integrins are exclusively expressed on leukocytes (59). The β2 integrin family contains four members: αLβ2 (lymphocyte function-associated antigen [LFA]-1 or CD11a/CD18), αMβ2 (Mac-1, CR3, or CD11b/CD18), αXβ2 (CR4, p150,95 or CD11c/CD18), and αDβ2 (CD11d/CD18) (60). LFA-1 and Mac-1 are ligands for ICAM 1, which is upregulated at sites of inflammation and facilitates transcellular diapedesis (56).

As β2 integrins are restricted to leukocytes, it has been assumed that carcinoma tumor cells must use different receptors or mechanisms for their adhesion to the endothelium at a potential colonization site (56). Adherence of tumor cells to platelets or neutrophils for use as a “linker” to enable firm adhesion has also been described (56). However, transcriptomic analyses of fusion hybrids suggest that tumor cell fusion with macrophages may result in acquisition of expression of β2 integrins. Indeed, macrophage-tumor cell fusion hybrids isolated from patients with pancreatic adenocarcinoma expressed transcripts for the β2 integrin subunit (*ITGB2*) (11). Macrophage-lung cancer hybrids also expressed the β2 integrin subunit (*Itgb2*), and at levels similar to that of unfused monocytes (10). Interestingly, hybrids also gained expression of CR4/CD11c (integrin alpha X, *Itgax*), albeit to a lesser degree than unfused monocytes. While *Itgax* was also expressed at a very low level by the parent lung cancer cell line, *Itgb2* was not detected, demonstrating acquired expression of a leukocyte-specific gene by hybrids (10).

Fusion with macrophages may also induce upregulation of non-leukocyte restricted cell adhesion molecules implicated in metastasis. VLA-4 (Very Late Antigen-4, or integrin α4β1) is the primary ligand for vascular cell adhesion molecule -1 (VCAM-1), a leukocyte adhesion molecule whose normal expression is restricted to endothelial cells and subpopulations of bone marrow cells (61). VLA-4 is the major integrin mediating firm adhesion in monocytes, particularly during inflammatory responses. VLA-4 is not a leukocyte-restricted antigen and has been implicated in metastasis of a variety of human cancers (61). However, expression of *Itga4* was upregulated in macrophage-colon cancer hybrids by nearly 3-fold above that of the parental MC38 cell line (9). Thus, fusion of tumor cells with macrophages may also increase the likelihood of successful extravasation by increasing expression of innate inflammatory signaling pathways known to facilitate metastasis.

There are myriad roles for cell adhesion molecules in metastasis, and several ligand-integrin combinations undoubtedly contribute to organotropism. It is also certainly theoretically possible for tumor cells to gain expression of leukocyte-specific integrins through processes such as genomic instability or uptake of extracellular vesicles bearing β2 integrins or their transcripts (13). However, it is noteworthy that monocytes and macrophages express all four members of the β2 integrin family, and that β2 integrins are implicated in macrophage fusion (62). Thus, fusion of tumor cells with monocytes/macrophages could provide an efficient mechanism to acquire expression of β2 integrins, and thereby increase the likelihood of arrest and extravasation at sites of inflammation such as those seen in premetastatic niches.

## 5 Discussion

Modern medicine has come a long way in developing therapies to successfully treat cancers that are localized or exhibit only regional spread, yet survival rates remain considerably lower in patients with distant metastases (**Table 1**). Even patients with cancers believed to be early-stage (Stages I-III) may eventually experience local relapse or recurrence in distant organs, potentially due to cancer cells that disseminated early in tumor formation and were undetectable at the time of diagnosis. For example, while 90% of breast cancer diagnosis are early-stage (63), approximately 1 in 6 women with node-positive HR+/HER2- early-stage BC receiving endocrine therapy will experience recurrence or death within 5-years of initiating treatment (64). Increasing our understanding of how cancer cells gain metastatic potential, which subpopulation(s) will metastasize and when, and/or why certain cancers disproportionately spread to particular organs may allow us to target these cells more effectively and improve patient outcomes.

**Figure 2:**
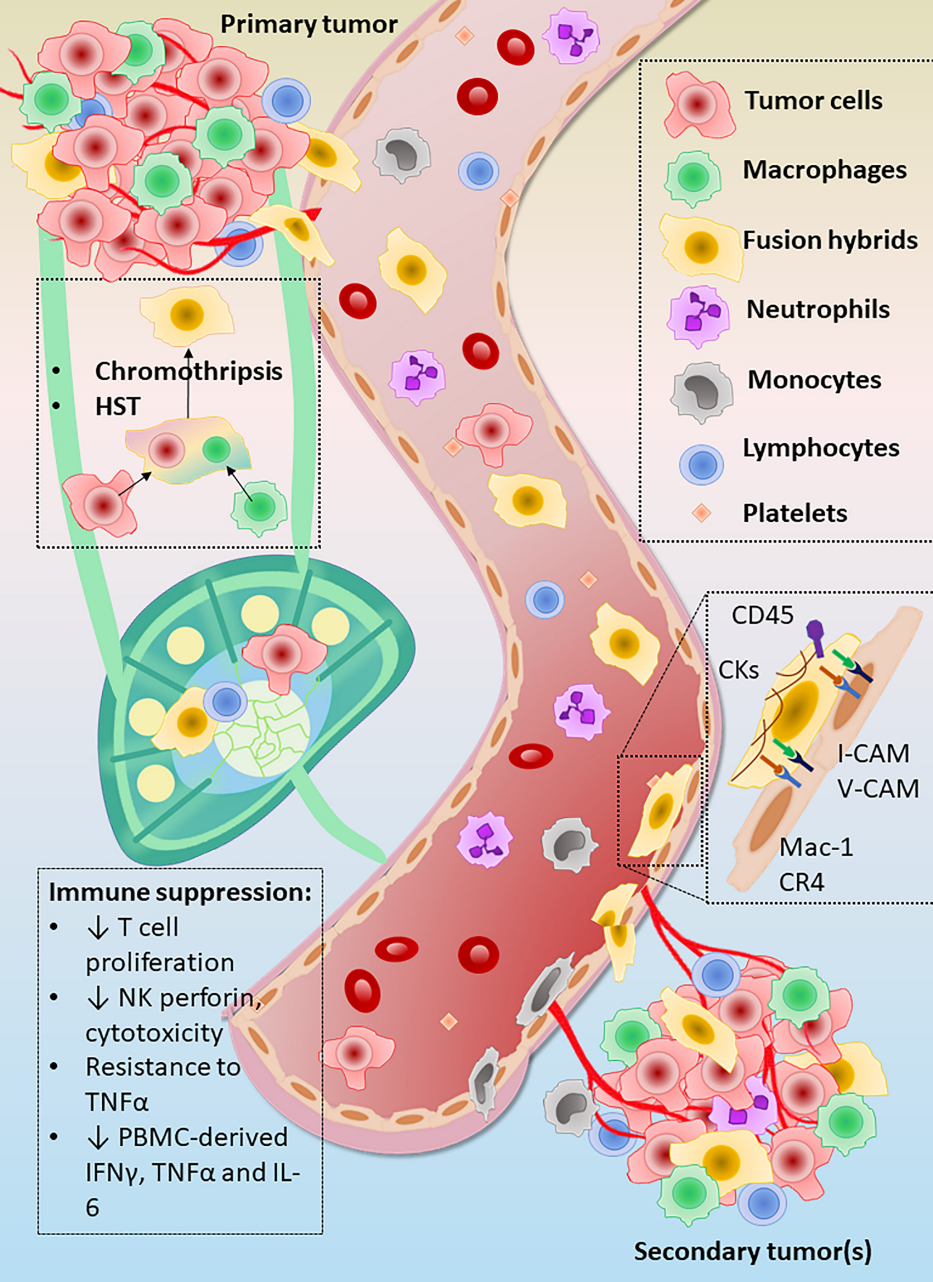
Summary of evidence for metastatic capacity in fusion hybrid cells. Heterotypic fusion between macrophages (green) and tumor cells (pink/red) followed by heterokaryon-to-synkaryon transition (HST) produces highly invasive and migratory fusion hybrid cells. These hybrid offspring are able to escape from the primary tumor microenvironment and greatly outnumber conventionally-defined circulating tumor cells (CTCs) in circulation. Fusion hybrids also show increased ability to inhibit immunosurveillance relative to unfused parent cancer cells, as shown by enhanced suppression of T cell proliferation and attenuation of perforin by natural killer (NK) cells. Production of tumor necrosis factor alpha (TNF-α, interleukin 6 (IL-6), and interferon gamma (IFNγ) by peripheral blood mononuclear cells (PBMCs) was also more strongly suppressed by fusion hybrids than by their respective parent cancer cell lines. A frequently used signature used for identification of CHCs is dual positivity for cytokeratins and the leukocyte identification marker CD45. Acquired expression of the typically leukocyte-restricted β2 family of integrin receptors, e.g., Mac-1 and CR4, by fusion hybrids may increase their affinity for sites of low-grade inflammation such as that seen in pre-metastatic niches.

Theories abound as to how a normal epithelial cell can undergo the genetic changes required for malignancy, many of which point to transformation through gradual and sequential accrual of individual driver mutations resulting in increasing genomic instability, tumor heterogeneity, and tumor evolution (65). However, malignant transformation may also occur via a “catastrophic genomic change” from a single event - homotypic and/or heterotypic cell fusion (19, 20, 22–24, 36, 38, 40, 66). While homotypic fusion between cancer cells remains difficult to detect, heterotypic fusion between macrophages and cancer cells appears to be a relatively common occurrence **(** **Figure 2** **)**, resulting in fusion hybrid cells with retained features of both parent cell types as well as new, unique expression patterns and cellular behaviors. Importantly, fusion hybrids show increased migratory and invasive behavior and appear to greatly outnumber conventionally-defined CTCs in circulation, further supporting fusion as a possible mechanism for acquisition of metastatic capability of tumor cells. Moreover, expression of leukocyte-restricted cell adhesion molecules by fusion hybrids could augment affinity for sites of inflammation, such as premetastatic niches, or even expand the number of locations at which cancer cells can extravasate. In light of the studies discussed in this review, fusion hybrids appear to be an important variable to consider in metastatic spread in general and in the etiology of metastatic organotropism observed in prevalent cancers.

Multiple studies defining macrophage-cancer cell fusion hybrids as cancer cells expressing the monocyte/macrophage lineage marker hemoglobin-haptoglobin complex scavenger receptor, CD163, have demonstrated a significant decrease in overall survival and distant recurrence-free survival in patients with greater numbers of CD163-positive tumor cells in their primary tumor (34, 67). Interestingly, preoperative irradiation in patients with colorectal cancer was also significantly associated with the presence of CD163+ tumor cells, suggesting a possible connection between X-rays and induction of cell fusion (34).

CHCs have also been shown to correlate with survival. Gast et al. conducted survival analysis on patients with pancreatic ductal adenocarcinoma to determine whether number of circulating tumor-immune hybrids cells and/or unfused circulating tumor cells correlated with disease stage, risk of death, or time-to-death in this population (9). In this study, the number of circulating CD45+/pan-cytokeratin(CK)+ fusion hybrids directly correlated with advanced disease and inversely correlated with overall survival. Specifically, patients with circulating CD45+/pan-CK+ cells above the median had a statistically significant increased risk of death (log-rank test, *P* = 0.0029, hazard ratio of 8.31), while number of CD45-/pan-CK+ cells did not correlate with either stage or survival. Similar to other studies discussed in previous sections, CD45-/pan-CK+ cells in this study were detected at quantities an order of magnitude lower than that of hybrid cells in circulation (9, 13). In light of the evidence previously discussed suggesting a less robust antitumoral immune response (9, 10), this observed magnitude difference between hybrids and conventionally-defined CTCs n peripheral blood could further support evasion of immunosurveillance by hybrids.

Interestingly, the proportion of fusion hybrid cells in human tumors varies greatly between different types of cancer, suggesting that certain types of cancer may be more or less amenable to cell fusion (24, 40). Activation state of the partner macrophage may also influence both likelihood of fusion and phenotype following HST/ploidy reduction. However, *in vitro* and *in vivo* analyses of macrophage activation have demonstrated a staggering number of macrophage activation phenotypes based on factors including the current cytokine milieu, oxygen tension, and proximity to tumor cells, among others (68, 69). Given the extent of macrophage diversity in tumors, future studies should investigate macrophage activation state in macrophage-cancer cell fusion and whether this variable also contributes to heterogeneity in the resulting fusion hybrid clones.

Profound heterogeneity among hybrid cells as well as inconsistencies in results across fusion studies further underscore the complexity of this research question. Multiple studies have demonstrated that macrophage-cancer cell fusion can also give rise to tumor hybrid cells exhibiting lesser or no metastatic capacity when compared to the parental cancer cell line (35, 70). Given the extensive heterogeneity observed among fusion hybrid cells, if fusion hybrids do in fact have an increased metastasis-initiating capacity, investigation is needed into the which characteristics are retained or gained in the PHSP that allow for this behavior. Additionally, while not discussed in previous sections of this review, conversion to a more epithelial cell phenotype following extravasation (mesenchymal-to-epithelial transition, MET) has been shown to be an important process in the formation of macrometastasis and metastatic colonization (71). How fusion hybrids might undergo MET is perplexing.

Despite acknowledged inconsistencies and limitations, analyses of possible correlation between fusion hybrids and cancer outcomes support the importance of tumor cell-myeloid cell nuclear fusion in cancer morbidity and mortality. Indeed, further research into the fascinating phenomenon of heterotypic cell fusion and the roles of fusion hybrids in human cancer biology is sorely needed. We look forward to future analyses revealing how a fusion partner is selected, which of the two cell types initiates fusion, and what the cues may be that begin the fusion process. Should fusion of cancer cells with macrophages underlie the phenomenon of metastatic organotropism, these studies may provide critical insight into therapeutic options to more effectively treat patients at risk for development of metastatic cancers.

## Author contributions

AJC was the primary contributor for writing, reviewing, editing, and visualization. SDH and MFC additionally contributed to reviewing, editing, and visualization. All authors contributed to the article and approved the submitted version.
